# Report of an Outbreak of Fever among the Crew of the Ottoman Line-Of-Battle Ship, Tesherefieh

**Published:** 1858-04

**Authors:** J. N. Radcliffe

**Affiliations:** late of the Ottoman Medical Staff.


### REPORT OF AN OUTBREAK OF FEVER AMONG THE CREW OF THE OTTOMAN LINE-OF-BATTLE-SHIP TESHEREFIEH;

### ALSO A REPORT OF AN OUTBREAK OF SCURVY AMONG THE CREW OF THE OTTOMAN BRIG-OF-WAR FEZRI-SEFET.

By J. N. RADCLIFFE, M.R.C.S.Eng., late of the Ottoman Medical Staff.

In the month of March, 1856, an outbreak of fever occurred among the crew of the Ottoman line-of-battle-ship *Tesherefieh*, at anchor off Sinope. The determining causes of the outbreak were apparently as follow.

After a period of weather during which the thermometer ranged in the shade from 36° to 64° Fahr., and the barometer ranged from 29·76 to 30·33; the winds being light, and from the south-east, east, or north-east, on the 4th of March the barometer sank to 29·68, and a heavy gale set in from the north-west, accompanied by rain, hail, and snow; and during the night the thermometer fell below the freezing point. The gale continued until daylight on the 5th, and the wind blew heavily, and snow fell throughout that day, the temperature ranging from 30° to 33° Fahr. On the 6th, the north-west wind having ceased during the preceding night, a slight south-west wind blew, and the temperature rose to 44°; but from the 7th to the 10th, northerly winds prevailed, accompanied by snow-showers, the temperature ranging, in the shade (between 9 A.M. and 10 P.M.), from 32° to 39° Fahr., and the barometer from 29·80 to 30·20.

Whilst the snow-storm continued, those of the crew of the *Tesherefieh* who slept upon the second deck suffered considerable discomfort from the cold; and they requested permission of the officers to sleep upon the lower deck. The request was granted; and for several nights the whole of the crew, numbering 540 men (about half the ship's complement), were huddled together on the confined and ill ventilated lower deck. It was while the crew were thus crowded together that disease broke out among them; and, in twenty days, eighty of the men were laid up with fever or bronchitis.*

Previous to this general outbreak of disease, there had

* The captain of the *Tesherefieh* had his own method of accounting for the outbreak of disease among his crew. According to him, it would appear that H.H. Omar Pacha has no great affection for, or opinion of, the Ottoman navy; and the captain suspected that, when the Pacha visited Sinope on the 7th of March, 1856, he cast the *evil eye* upon the *Tesherefieh*, and that, in consequence, disease broke out on board the vessel.

been two or three cases of continued fever among the crew; but, with these exceptions, the men had had good health, and they had been well fed. The number of sick among the crew was said to vary ordinarily from five to fifteen.

On the 20th of March, when eighty of the men had been attacked with sickness, and when fresh cases of illness were occurring daily, the majority of the sick were removed from the ship and sent to the quarantine station.

On the 24th, the captain of the *Tesherefieh* sought aid of Dr. Le Mesurier, acting deputy inspector of hospitals, attached to the Ottoman army; and Mr. H. W. Kiallmark and myself were directed to take charge of the patients in the quarantine station.

Up to this period, the sick had been attended by the surgeon of the ship; and five cases of *fever* had terminated fatally.

The quarantine buildings of Sinope are situated in the lower quadrangle of the ancient and ruined citadel. They consist of a large two-storied building and several out-buildings. The large building is of an oblong form; it is rough-cast and white externally, and the roof is tiled. It has six rooms on the lower floor, and seven on the upper, the corridors running in the rear of the building; and from each extremity projects, also in the rear, a small wing containing the privies. The rooms, with one exception, are about fourteen feet square, and eleven feet high, the floors and ceilings being of wood, and the walls of plaster whitewashed. One room, on the upper floor, is of much smaller dimensions. The rooms are lit with one, two, or three glazed windows, the sashes of which open on longitudinal axes. The privies are floored with stone slabs, which have a simple aperture near the centre (after the Turkish fashion), the aperture communicating with a cylindrical earthenware tube. This tube opens out into a square drain, and the drain terminates in a large open circular cesspool, which is situated about twelve feet distant from the south-west angle of the building. The cesspool has a diameter of about twelve feet; it is lined with rough masonry, and the depth to the surface of the contents is about ten feet.

Beneath the walls of the quadrangle are several sheds, adapted for kitchens, store-rooms, and quarters for the guard; and there are several wells within the area, but the water is used only for cleansing purposes. The principal portion of the area of the quadrangle is occupied by rough hillocks, the remains of ruined buildings; and, in the summer and autumn, a rank vegetation covers the surface. Conspicuous amid the hillocks and vegetation, and contiguous to one of the sheds, is a huge cavity, which forms a somewhat striking illustration of the second gulf in Malebolge, for—

> "Upon the banks a scurf,
> From the foul steam condensed, encrusting hangs,
> And holds sharp combat with the sight and smell."

Over this gulf is perched, very ingeniously, a detached privy. Two or three tall trees occupy a corner of the quadrangle; a dried-up fountain stands apart; and here and there ivy straggles up the walls.

Certainly the situation of the quarantine buildings is picturesque. The varied area, the tall, rugged, and time-worn walls and towers, and the wealth of antiquity built into them, are grateful to the eye; and the odds and ends of sculpture, the fragments of inscriptions to the Antonines and the Cæsars, the faint Byzantine letterings, and the slabs covered with richly florid Turkish characters—these stimulate the mind. Moreover, from the summits of the walls and towers, as lovely a prospect may be beheld as the heart of man could desire.

Notwithstanding that these things might prove of interest to the quarantine prisoner or patient, the buildings are badly situated. The high walls shut out the winds; and, except by the sea-gate, there is no mode of drainage, and through that portal a sluggish filthy stream of offscourings meanders, polluting the surface of the ground. The doors and windows of the different buildings have also been used as the readiest outlets for ejecting "slops"; and, in consequence, the surface of the ground immediately contiguous to the buildings is covered with a thick layer of decomposing organic matter. To these detractions have to be added the huge open cesspools, and the rank verdure.

The quarantine buildings had been set apart for a military hospital soon after the removal of the Ottoman army, under the command of H.H. Omar Pacha, to Mingrelia; and, under the superintendence of Dr. Le Mesurier, the different buildings had been so cleansed and fitted up as to form, notwithstanding their bad position, a very satisfactory temporary hospital.

It was while the buildings were thus fitted for hospital purposes that the sick from the *Tesherefieh* were removed to them, and that Mr. Kiallmark and myself assumed the charge. On the day when we entered upon our duties, we found forty-three patients in the main building, and six patients in an adjoining shed. The sick in the main building, with the exception of one patient, wildly delirious, who was secured in a room apart, were packed in six of the rooms, seven patients being placed in each of the six apartments. The beds, which were laid upon the floor, touched each other, being arranged side by side; and there was barely sufficient space left for the door to open, and for a stove to burn wood, or for a *mangal* (charcoal burner). The stoves were fully heated, and the *mangals* were filled with incandescent charcoal, and almost every crevice by which air could be admitted to the rooms was closed up. The state of the atmosphere, under these circumstances, may be better conceived than described; the intense heat, the pungent wood-smoke, or the unpleasant smell from the burning charcoal, and the foul odour from the patients, were almost overpowering. The state of the patients was in keeping with the condition of the wards. Two of the men, suffering from fever, were dying; and the remainder, without exception, were so depressed, and the majority of the cases had so formidable an aspect, that Mr. Kiallmark and myself were almost inclined to imagine that the fever, the most prevalent affection, was, as common report has it, of a malignant character. We immediately caused the patients to be distributed through the different wards and sheds, and took steps to secure an efficient ventilation; and in thirty-six hours there was so marked an improvement in the state of the cases, that it could not be doubted that the peculiar depression, and the more formidable symptoms which had characterised the cases when first seen, had depended, in a great measure, on the crowded condition and the imperfect ventilation of the wards.

In the ventilation of the sheds, recourse was had to a principle which has been very ably utilised by Mr. Charles Watson, of Halifax. If a chamber containing air of a higher temperature than the external atmosphere, communicate with the outer air by means of a tube which has a diaphragm running along the centre, a double current will be established, the warmer air passing out of the chamber along one division of the tube, the colder air passing into the chamber along the other division; and, if the tube be of fitting dimensions, an amount of fresh air will pass into the chamber sufficient to keep it well ventilated, even if there be no other means of ingress for the air. A wooden turret, with central diaphragm, and properly roofed to shut out rain, was run from the ceiling of each shed through the roof, and by this means a tolerably good ventilation was secured, even under unfavourable circumstances. The value of this method of ventilation was great, as it could not well be tampered with, and the Ottoman has a most pestiferous habit of closing every aperture which will admit air, during cold or boisterous weather. In the main building, where this system of ventilation could not be adopted, it was only by constant watchfulness that a proper ventilation could be secured.*

Mr. Watson's ingenious application of this principle of ventilation to public buildings and dwelling-houses is well worthy of attention.

After placing the patients in the best position that our resources would admit of, our next efforts were directed to the well being of the ship and crew. On an inspection, we found that the ship was tolerably clean, and that there was an absence of foul smells. The upper deck was without guns, and we recommended that the whole of the crew should, for a fortnight, sleep upon that deck; and that, after a thorough cleansing by water, the decks should be cleansed by dry-rubbing for several weeks; that pannikins filled with charcoal should be distributed about the lower deck; and that all cases of sickness should be sent on shore as soon as they occurred. These measures were carried out, and in a fortnight fever ceased to appear on board the ship.

The *Tesherefieh* is an American built ship, and she was, at the time of the outbreak, three years old. She carries ninety-six guns, but during the war she was used as a troop ship, and she was not fully manned. The crew, of whom 480 were drawn up for inspection, had not a very satisfactory appearance. The men were wretchedly attired; and, as a rule, they were inferior in stature and physical development to the bulk of the soldiers under the command of H.H. Omar Pacha. This remark, moreover, holds good of the crew of the brig-of-war *Fezri-sefet* also, at that time anchored off Sinope, notwithstanding that there were a greater proportion of well developed men among the crew of that vessel, than among the crew of the *Tesherefieh*.

Twenty-nine of the crew of the *Tesherefieh* were found to be suffering from incipient fever, or from other diseases; and in the examination of the *tongues* of the crew, the interesting fact was ascertained, that, of the whole number examined, 480, only twenty were in a truly healthy condition. The remainder were either pale, flabby, and broad, the edges being indented with the teeth; or they were pointed, red,

* The use of the *mangal* is at the best a very unsatisfactory method of heating an apartment. The charcoal is ignited in the open air, and, when fully incandescent, it is brought into the apartment.

and glazed, or pale and fissured. Few, also, of the *Fezri-sefet's* crew were found to possess healthy tongues. At an inspection of the crew of that vessel, 120 in number, it was ascertained that, with two or three exceptions, the tongues of the men were pale and flabby, or more or less fissured. During a final inspection of the crew of the *Tesherefieh,* at which Dr. Le Mesurier was present, he remarked that he had observed a similar peculiarity of the tongue to prevail among the rice-eating Hindoos. I had no opportunity of ascertaining whether a like condition of the tongue was prevalent among the soldiers of the Ottoman army.

The *rations* of the Ottoman sailor, except when at sea, are similar in quality and quantity to those of the Ottoman soldier; but, when at sea, the sailor receives, in lieu of meat, a ration of from 50 to 60 dihrems (5 to 6 ounces) of fresh olives daily. The olives are eaten with vinegar, or oil and raw onions.

The daily ration scale of the men, both in the army and navy, is as follows:—

*Bread,* 31 oz. (that is 300 dirhems, the *oke* containing 400 dirhems); *meat,* $10\frac{1}{2}$ oz. (92 dirhems), except on two days of the week, when none is issued; *rice,* $2\frac{3}{4}$ oz. (25 dirhems), which is used in soup, but on the two days when no meat is issued, 92 dirhems of rice are substituted for it, the rice being cooked as *pillaff; sheep's tail fat,* $1\frac{3}{4}$ oz. (16 dirhems); *salt,* $\frac{1}{2}$ oz.; *vegetables* (onions, *nohad* (chick-pea?), haricot beans, etc.), a variable quantity.

The crew of the *Tesherefieh* were well fed and well cared for by the captain, and this even under circumstances of considerable difficulty in procuring the necessary supplies of food. The crew of the *Fezri-sefet* were less carefully attended to. That vessel had been stationed off Kamiesch ninety days; and, during that period, the crew had rarely received rations of meat and vegetables. Soon after the brig was stationed at Sinope, several of the men were prostrated from scurvy, and they were sent into the hospital; and, on an inspection of the crew, it was found that every man was affected, more or less, with the disease. The ship's stores contained neither lime-juice nor citric acid, nor the mineral acids, and the apothecary was helpless among the crew.

The only vegetable food which could be procured in Sinope, in quantity sufficient for the men, when the vessel anchored off the port (March), was onions; and it was directed that this vegetable should be issued freely to the crew daily, and eaten raw. A scanty supply of fresh meat was also obtained; but no other measures for checking the disease were adopted, as the captain of the brig seemed to be averse to receiving assistance from the English surgeons. The change in the diet was, however, sufficient to prevent any further development of the scurvy while the ship remained at Sinope.

The *number* of the sick received into the hospital from the two ships, the *character* of the cases, and the *mortality*, were as follows:—

From the 24th of March to the 29th of April, 103 cases were admitted into the hospital. Of these, 57 were cases of *fever*, 53 being cases of *continued*, and 4 cases of *intermittent fever*. Of the remaining cases, 11 were cases of *diarrhœa*, 10 of *bronchitis*, 4 of *conjunctivitis*, 8 of *scurvy*, 11 of various *chronic diseases*, 1 was a case of *dysentery*, and 1 of *pleuro-pneumonia*. The whole of the cases of scurvy, and two cases of diarrhœa, came from the *Fezri-sefet*.

The total number of *deaths* amounted to five; three being deaths from continued fever, one being a death from diarrhœa (a chronic case), and one from scurvy.

The continued fever was of an asthenic type; the majority of the cases were complicated with bronchitis; and the treatment had to be directed mainly towards the sustentation of the vital powers. The other forms of disease presented nothing unusual in their progress.

